# Considerations for Clinical Therapeutic Development of Statins for Neurodevelopmental Disorders

**DOI:** 10.1523/ENEURO.0392-19.2020

**Published:** 2020-03-02

**Authors:** Myrthe J. Ottenhoff, Lianne C. Krab, Ype Elgersma

**Affiliations:** 1 Department of Neuroscience, ENCORE Expertise Center for Neurodevelopmental Disorders, Erasmus Medical Center, 3015 GD, Rotterdam, The Netherlands; 2 Cordaan Outpatient Clinic for ID Medicine, 1033 PK, Amsterdam; 3 Odion Outpatient Clinic for ID Medicine, 1531 AD, Wormer; 4 Department of Pediatrics, Amsterdam UMC, 1105 AZ, Amsterdam, The Netherlands

**Keywords:** autism spectrum disorder, cognitive trials, fragile X, lovastatin, RASopathies, simvastatin

## Significance Statement

The 3-hydroxy-3-methyl-glutaryl-coenzyme A reductase (HMG-CoA reductase) inhibitors lovastatin and simvastatin have both been investigated in clinical trials designed to treat the cognitive deficits associated with neurodevelopmental disorders such as neurofibromatosis type 1, fragile X and autism. In a recent study, the therapeutic efficacy of lovastatin and simvastatin were compared in a fragile X (*Fmr1*) mouse model. The authors concluded that lovastatin was superior to simvastatin in rescuing the *Fmr1* phenotypes, and cautioned against considering simvastatin as treatment for neurodevelopmental disorders. We discuss these findings in the context of published literature and argue that more support is needed for this potentially far-reaching conclusion. We further provide recommendations to improve the translation of pre-clinical studies of cognitive disorders into the clinical domain.

## 

The potential use of statins for antagonizing RAS (rat sarcoma viral oncogene homolog) signaling was first recognized nearly three decades ago (Mendola and Backer, 1990; Sebti et al., 1991). Functional RAS requires post-translational farnesylation to become membrane bound and active. Since farnesyl (like cholesterol) is a product of the mevalonate synthesis pathway, its synthesis can be reduced by interfering with the rate-limiting enzyme, 3-hydroxy-3-methyl-glutaryl-coenzyme A reductase (HMG-CoA reductase). Statins, designed as high-affinity HMG-CoA reductase inhibitors, are commonly prescribed for hypercholesterolemia. Over the past several decades, various types of statins have been extensively investigated as potential cancer therapeutics using cellular models, mouse studies, and human clinical trials (Gazzerro et al., 2012; Pisanti et al., 2014; Sopková et al., 2017). On the basis of these findings, Alcino Silva and colleagues explored whether statins might have efficacy in the treatment of RASopathies, a group of neurodevelopmental disorders resulting from mutations that lead to overactivation of the RAS/extracellular signal-regulated kinase (ERK) signaling pathway. Specifically, it has been shown that lovastatin can ameliorate the cognitive deficits in animal models of neurofibromatosis type 1 (*Nf1* mice) and Noonan syndrome [*Ptpn11* (protein tyrosine phosphatase non-receptor type 11) mice; Li et al., 2005; Lee et al., 2014], although it failed to rescue the deficits in a mouse model of Costello syndrome (*Hras* mice; Schreiber et al., 2017).

To translate the mouse findings to the clinic, statins were tested in several randomized placebo-controlled trials aimed at improving cognitive function (Krab et al., 2008; Van Der Vaart et al., 2013; Bearden et al., 2016; Payne et al., 2016; Moazen-Zadeh et al., 2018; Stivaros et al., 2018). These trials used either the first commercially-available statin, lovastatin, or a second generation statin, simvastatin, that is highly similar in structure and pharmacokinetics to lovastatin (Neuvonen et al., 2008; Gazzerro et al., 2012). Notably, simvastatin has not been used in animal models of the RASopathies, but like lovastatin, simvastatin has been shown to decrease ERK signaling in cultured cells (Fürst et al., 2002; Guillén et al., 2004; Miura et al., 2004; Ghittoni et al. 2005, 2006; Khanzada et al., 2006; Ogunwobi and Beales, 2008; Sundararaj et al., 2008; Kang et al., 2009), as well as *in vivo* (Chen et al., 2010; Lee et al., 2011; Takayama et al., 2011), including the brain (Ghosh et al., 2009). Simvastatin has a 2- to 4-fold increased potency against HMG-CoA reductase and a higher blood-brain barrier permeability compared with lovastatin (van Vliet et al., 1996; Sierra et al., 2011; Gazzerro et al., 2012; Fong, 2014). These comparative properties of simvastatin and lovastatin might suggest at minimum the non-inferiority of simvastatin versus lovastatin. Although both lovastatin (Mainberger et al., 2013; Bearden et al., 2016) and simvastatin (Stivaros et al., 2018) showed some potential benefits in smaller trials for NF1, three independent large randomized controlled trials of cognition and behavior in children with NF1 using a dose of 40 mg/d of simvastatin (Krab et al., 2008; Van Der Vaart et al., 2013) or lovastatin (Payne et al., 2016) failed to show efficacy in the primary outcome measures, even when treatment was administered for one year (Van Der Vaart et al., 2013). Thus, no benefits (nor meaningful differences) have been observed between simvastatin and lovastatin in treating NF1-associated cognitive dysfunction. The only sufficiently powered trial that has suggested a benefit for statin treatment on behavior came from a recent study on children with ASD, in which simvastatin was used adjunctively, which yielded a significant decrease of irritability and hyperactivity, but no improvement on three other scales of a behavioral checklist (Moazen-Zadeh et al., 2018).

In light of these mostly negative findings, it is crucial to try to understand why these clinical trials failed. For that, more research is required, and the study by Muscas et al. (2019) is a very important step in that direction. In this study, the authors compared lovastatin with simvastatin treatment in an animal model of fragile X (*Fmr1* (fragile X mental retardation) mice). Although ERK signaling in *Fmr1* mice is not increased under baseline conditions, it has been shown that the ERK pathway in these mice is hypersensitive and contributes to the excessive protein synthesis which is considered one of the core mechanisms underlying fragile X syndrome pathophysiology (Osterweil et al., 2010). Moreover, lovastatin treatment rescues the ERK-dependent increased of protein synthesis as well as the sensitivity to audiogenic seizures of *Fmr1* animals (Osterweil et al., 2013). Given that simvastatin is a more potent inhibitor of HMG-CoA reductase than lovastatin, one would expect that simvastatin treatment would result in a better, or at least a similar rescue. However, in the recent study, Muscas et al. (2019) surprisingly concluded that lovastatin is superior over simvastatin in reducing ERK activation, as well as in its ability to rescue the downstream phenotypes of ERK activation: increased protein synthesis and sensitivity to audiogenic seizures. Therefore, the authors caution against the assumption that simvastatin is a suitable substitute for lovastatin with respect to the treatment of fragile X or other neurodevelopmental disorders.

If correct, this conclusion would have far reaching implications. Given the increased potency of simvastatin to reduce HMG-CoA reductase, it would suggest that the previously demonstrated rescue of RASopathy phenotypes by statins is not mediated by attenuation of RAS farnesylation but rather through an unknown mechanism that is absent or less potent for simvastatin. This would have considerable impact in the design of potential future clinical trials for treatment of cognitive deficits in RAS related disorders. However, in reviewing the study of Muscas et al. (2019), the question arises whether the study truly represents a side-by-side comparison that warrants such a strong conclusion. Most notable, there is no experiment in which lovastatin and simvastatin are compared at the same dose (and with the same vehicle). In addition, a statistical analysis that would enable a direct comparison of lovastatin and simvastatin is lacking.

Given the aforementioned large body of literature that shows that simvastatin can reduce RAS/ERK signaling in cultured cells as well as *in vivo*, the finding by Muscas and colleagues, that simvastatin (in contrast to lovastatin) fails to reduce ERK signaling in brain slices, is quite remarkable. However, it is important to note that the investigators used 50 μM lovastatin but a 100- to 500-fold lower dose of simvastatin (the maximum used simvastatin dose is 0.5 μM). Importantly, the authors previously showed that a lovastatin dose of 10 μM is not effective in this particular assay (Osterweil et al., 2013), hence, the failure of simvastatin to reduce ERK activation at doses far below that is not entirely surprising.

For the protein synthesis experiments (which is sensitive to increased ERK signaling), the investigators used again a much lower dose of simvastatin (10- to 500-fold lower) compared with lovastatin (the maximum used simvastatin dose is 5 μM). The lack of efficacy at such a low dose of simvastatin is again not entirely surprising, as the authors previously showed that the lovastatin dose needs to exceed at least 10 μM to be effective in this assay (Osterweil et al., 2013). An elegant study by Tuckow et al. (2011) showed that 10 μM simvastatin is indeed able to reduce protein synthesis in a mevalonate dependent way, which indicates that at this dose (and under these conditions) there is a clear HMG-CoA-dependent effect of simvastatin on protein synthesis.

The most surprising finding of the study by Muscas and colleagues is the finding that simvastatin treatment at low dose actually worsened the *Fmr1* phenotype by further increasing protein synthesis rates. This effect was found to be independent of ERK signaling. This aspect of the study is not only a noteworthy finding, it is also a very worrisome finding with respect to fragile X clinical trials, where the overarching goal is to use statins to reduce protein synthesis and thereby rescue the behavioral phenotypes (Çaku et al., 2014). For the follow-up of these trials it would be of great importance to know if a comparable (low) dose of lovastatin (below the dose needed to inhibit ERK) would have a similar negative effect on this phenotype, especially since the dose that can be safely used in clinical trials is much lower than the *in vivo* dose used in this study.

Whereas in the large, placebo-controlled clinical trials lovastatin was used at the same dose as simvastatin, Muscas and colleagues used a 2- to 30-fold lower dose of simvastin than the dose used for lovastatin (100 mg/kg) for their *in vivo* epilepsy experiments. Importantly, the authors previously showed that reducing the lovastatin dose to 30 mg/kg, only rescues the seizure phenotype of *Fmr1* mice in certain mouse strains (i.e., inbred C57BL/6; Osterweil et al., 2013), indicating that also for lovastatin a lower dose than 100 mg/kg may not always be effective in this assay.

Beside these differences in dosing, it is questionable if the overall experimental design justifies the conclusion that lovastatin is superior over simvastatin to rescue the core phenotypes of *Fmr1* mice. If the ultimate goal of the study is to directly compare two drugs with each other, the drugs should not only be tested side-by-side as interleaved experiments, they should also directly be compared with each other using a statistical analysis that tests for a main effect of treatment, and if significant, followed by a *post hoc* analysis to compare the drugs. That this can have a substantial effect on the conclusion, can be illustrated by reanalysis of the dichotomous audiogenic seizure data from the paper of Muscas et al. (2019). Performing such analysis using a logistic regression model, reveals that there is a significant main effect of genotype (χ^2^(4) = 51; *p* < 0.0001), no effect of vehicle (χ^2^(2)=0.3; *p* = 0.9) and no interaction of vehicle and genotype (χ^2^(1) = 0.2; *p* = 0.7). These are important control measures since different concentrations of DMSO solvent were used for each drug and could potentially affect the outcome on seizures (Carletti et al., 2013). This analysis further shows a trend for a main effect of treatment (χ^2^(6) = 12; *p* = 0.07), but not for the interaction between genotype and treatment (χ^2^(4) = 4; *p* = 0.3). When performing a *post hoc* Tukey’s test, neither the *Fmr1*-lovastatin versus *Fmr1* “low dose” of simvastatin (*p* = 0.96) nor the *Fmr1*-lovastatin versus *Fmr1* “high dose” of simvastatin treatment (*p* > 0.99) are significantly different from each other. Hence, despite the fact that the lovastatin dose was 2- to 30-fold higher than simvastatin dose, it does not seem to perform significantly better than simvastatin in this seizure assay.

So how can the lack of efficacy of both lovastatin and simvastatin in prior randomized clinical trials of neurodevelopmental disorders be explained, and what can we learn from pre-clinical studies such as the Muscas et al. (2019) study? We believe that two factors are very important to consider when translating findings in animal models to clinical trials in humans.

**Figure 1.**
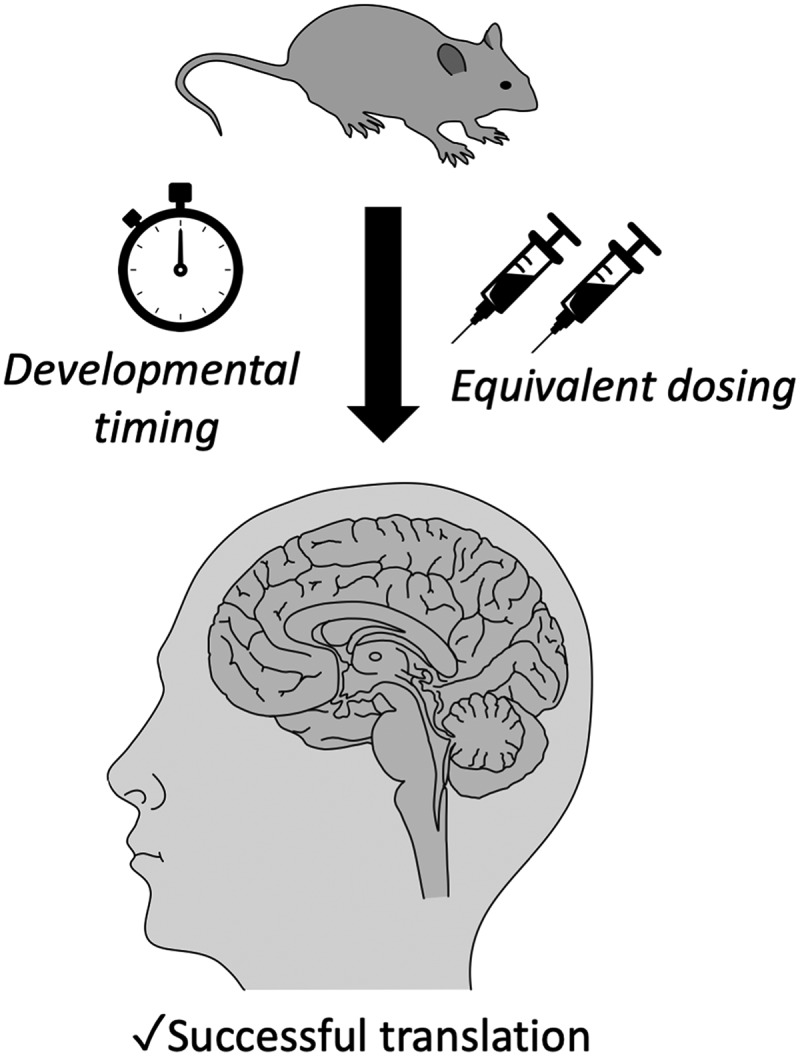
To ensure optimal translation of animal experiments to cognitive clinical trials, it is important that the drug treatment used in animal studies resembles that of clinical trials with respect to equivalent dose (considering also interspecies differences in pharmacodynamics and pharmacokinetics of target tissue), route of administration and drug similarity. Moreover, it is important to take into account the timing of drug administration, as treatment of neurodevelopmental disorders may require intervention during a critical window of development.

The first critical factor is the translation of dosing from mice to men. The dose in which a particular drug rescues a phenotype in animal model does not always translate into a clinically applicable and safe dose in humans (Figure 1). For instance, the study by Muscas et al. (2019) used a lovastatin dose of 100 mg/kg (intraperitoneal injection) for testing of audiogenic seizures in the fragile X mouse model. This dosing regimen is much higher than needed to inhibit HMG-CoA reductase (Van de Steeg et al., 2013), or the dose used for behavioral rescue in earlier studies of RASopathy mouse models (10 mg/kg, subcuteanous injection; Li et al., 2005; Lee et al., 2014; Schreiber et al., 2017). More importantly, it is ∼100-fold higher than the equivalent dose used in the clinical trials when also considering bioavailability for oral versus intraperitoneal injection (Zhu et al., 2011; dose conversion calculated by FDA guidelines; www.fda.gov/media/72309/download). Hence, although the partial rescue of audiogenic seizures is of compelling scientific interest, it is important to realize that the direct translational value of such high doses is limited. And although a rescue of behavioral deficits in *Fmr1*, *Nf1*, and *Ptpn11* animal models has been observed using an oral dose that more closely reflects the dosing used in clinical trials (Li et al., 2005; Osterweil et al., 2013; Lee et al., 2014; Asiminas et al., 2019), it still cannot be excluded that the effective dose of statins in the mouse brain is different from the human brain, as even small species differences in blood brain permeability could eliminate the beneficial effect of statins (Hoshi et al., 2013). This study by Muscas et al. (2019) underscores the importance of looking at effective dosing ranges, and more detailed (*in vivo*) pharmacological studies in animal models should be performed to elucidate the dose dependency of therapeutic benefit.

The second factor that may affect successful translation to patients is the timing of drug administration. Whereas most pre-clinical studies involved drug treatment of adult animal models of neurodevelopmental disorders, it is conceivable that this may not be effective in human patients and that treatment of patients should be started in young children to be maximally effective. Conversely, if a behavioral rescue is observed in young mice (e.g., the rescue of seizures in *Fmr1* mice was performed on postnatal day (P)18–P29 mice; Osterweil et al., 2013; Muscas et al., 2019), it is important to investigate if such a rescue is still observed when the brain has fully matured. Interestingly, a recent study in a rat model of fragile X syndrome demonstrated that adult *Fmr1* animals no longer exhibited cognitive deficits following brief lovastatin treatment at young age only (Asiminas et al., 2019). Such studies should be further exploited to delineate the precise critical period for optimal treatment of neurodevelopmental disorders (Silva-Santos et al., 2015).

Once these two critical parameters are known for both simvastatin and lovastatin, it may be warranted to consider new clinical trials of statins for treatment of cognition in neurodevelopmental disorders. Hopefully, when using the right conditions, statins will be as effective in humans as they were shown to be in multiple animal models.

## Synthesis

Reviewing Editor: Quentin Pittman, University of Calgary Decisions are customarily a result of the Reviewing Editor and the peer reviewers coming together and discussing their recommendations until a consensus is reached. When revisions are invited, a fact-based synthesis statement explaining their decision and outlining what is needed to prepare a revision will be listed below.

In this commentary authors, after reviewing basis for the use of statins in the treatment of RASopathies, they state their opinion on the potential interpretation of the preclinical data presented by Muscas et al, 2019 in eNeuro, with regard to the effects of simvastatin and lovastatin in a mouse model of fragile X syndrome. In this commentary authors discuss the dosages, experimental design and conclusions on Muscas et al, 2019 to conclude that there are other potential explanations that were disregarded in the commented eNeuro publication to explain their lack of effect of sinvastatin compared to lovastatin. Such potential discrepancies between sinvastatin and lovastatin discussed in the commentary may arise from lack of comparable doses or schedule/timing of the treatment intervention.

This commentary is useful for the neurodevelopmental disorders community because it gives some important background on simvastatin and lovastatin in mouse models and patients and provides perspective to the recently published Muscas et al 2019 paper. However, the reviewers think that the authors have not presented the Muscas findings entirely accurately and revisions should be made to provide a more informed or balanced view of the Muscas paper.

Major:

1. First in response to the criticism that lavastatin and simvastin are not directly compared to each other -Muscas et al. tested lovastatin and simvastatin in all the same assays in Fmr1 KO mice, protein synthesis rates, ERK phosphorylation and audiogenic seizures (and reproduced results from their previous lovastatin study). The authors of the commentary indicate that effects of simvastatin and lovastatin were not directly compared to each other and making a comparison of some audiogenic seizure phenotypes (clonic or tonic seizures) between lovastatin and simvastatin showed no difference. This analysis is not valid based on the experimental design of the Muscas et al study. Due to variability in clonic and tonic seizures across cohorts (or litters) (compare KO vehicle in Figure 4, C,F,I) the effects of a drug (simvastatin or lovastatin) are compared to vehicle-treated, KO littermates. Based on the description in Muscas et al. they used littermate controls injected with either vehicle or drug and found that lovastatin, but not simvastatin (at two doses) corrected several measures of AGS- incidence, severity and latency. In order to compare simvastatin to lovastatin directly, the study would need to be repeated with lovastatin, simvastatin or vehicle treatment in littermates or the same cohort, which was not done in the Muscas study. Because this comparison is not valid, the authors should remove text about the comparison of simvastatin and lovastatin directly on the clonic-tonic seizures. Instead the authors may suggest a direct comparison study.

2. In response to a)the criticism that the drugs were not compared at the same dose---Muscas et al tested a range of simvastatin doses (0.1-5.0 uM) and one effective dose of lovastatin (50uM) in the ERK and protein synthesis assays. The authors found a dose dependent increase in the protein synthesis rates at higher doses of simvastatin (0.5 and 5 uM) which could confound or preclude analysis of very high doses of simvastatin on these Fmr1 KO phenotypes. This should be mentioned by the authors of the commentary.

The fact that Muscas et al., did show effects of simvastatin on protein synthesis rates, albeit in a distinct direction that lovastatin, indicates the drug was effective. However, because simvastatin did not affect P-ERK at these doses suggests it may be acting through a distinct mechanism from lovastatin, perhaps as a result of its increased efficacy at HMG-CoA reductase or other off target effects. Or lovastatin has off target effects at higher doses on ERK and reduced protein synthesis. This could be considered by the commentary.

Minor:

1. In doing the estimation of the equivalent doses between human and mice, toward the end of the main text, authors should specify the method they used to perform the dose calculation in both species.

2. It would help to define concepts and gene names such as RAS, PTPN11, HRAS, HMG-CoA, etc.

3. While in another part of the text they mention Neurofibromatosis type I, and they could use NF1 instead.

## Author Response

In this commentary authors, after reviewing basis for the use of statins in the treatment of RASopathies, they state their opinion on the potential interpretation of the preclinical data presented by Muscas et al, 2019 in eNeuro, with regard to the effects of simvastatin and lovastatin in a mouse model of fragile X syndrome. In this commentary authors discuss the dosages, experimental design and conclusions on Muscas et al, 2019 to conclude that there are other potential explanations that were disregarded in the commented eNeuro publication to explain their lack of effect of sinvastatin compared to lovastatin. Such potential discrepancies between sinvastatin and lovastatin discussed in the commentary may arise from lack of comparable doses or schedule/timing of the treatment intervention.

This commentary is useful for the neurodevelopmental disorders community because it gives some important background on simvastatin and lovastatin in mouse models and patients and provides perspective to the recently published Muscas et al 2019 paper. However, the reviewers think that the authors have not presented the Muscas findings entirely accurately and revisions should be made to provide a more informed or balanced view of the Muscas paper.

Response: We thank the reviewer for the thoughtful comments. We realize that in our efforts to be succinct, we indeed lost some important details that should have been included. We have now significantly expanded the commentary part in which we discuss the Muscas et al finding. Specifically, we provided more background (needed for the reader unfamiliar to the Fragile X field), we more accurately/detailed summarized the findings and stayed closer to the actual wording the authors used for their conclusions. We have also been in touch with the authors and sent the first submitted as well as final submitted manuscript to them for further checking for factual errors and to make sure we had not misrepresented their conclusions.

Major:

1. First in response to the criticism that lavastatin and simvastin are not directly compared to each other -Muscas et al. tested lovastatin and simvastatin in all the same assays in Fmr1 KO mice, protein synthesis rates, ERK phosphorylation and audiogenic seizures (and reproduced results from their previous lovastatin study). The authors of the commentary indicate that effects of simvastatin and lovastatin were not directly compared to each other and making a comparison of some audiogenic seizure phenotypes (clonic or tonic seizures) between lovastatin and simvastatin showed no difference. This analysis is not valid based on the experimental design of the Muscas et al study. Due to variability in clonic and tonic seizures across cohorts (or litters) (compare KO vehicle in Figure 4, C,F,I) the effects of a drug (simvastatin or lovastatin) are compared to vehicle-treated, KO littermates. Based on the description in Muscas et al. they used littermate controls injected with either vehicle or drug and found that lovastatin, but not simvastatin (at two doses) corrected several measures of AGS- incidence, severity and latency. In order to compare simvastatin to lovastatin directly, the study would need to be repeated with lovastatin, simvastatin or vehicle treatment in littermates or the same cohort, which was not done in the Muscas study. Because this comparison is not valid, the authors should remove text about the comparison of simvastatin and lovastatin directly on the clonic-tonic seizures. Instead the authors may suggest a direct comparison study.

Response: The authors used as a key selling point that the study actually represents a direct side-by-side comparison, and the methods for audiogenic seizures explicitly states: ‘Test cohorts were counterbalanced for genotype and treatment. The experimenter was blind to drug and genotype’. That suggested to us that the experiments truly represented a direct comparison study, which should reduce the experimental variation indicated by the reviewer (frankly, the differences in vehicle treated animals are actually not that large).

We have been in touch with the senior author of the study (Emily Osterweil) to clarify this issue. She confirmed the experiment was designed as a side-by-side experiment. But she indicated they had used different doses of DMSO for both drugs, and that they were not aware of a good statistical measure for dichotomous outcome measures to allow for a direct comparison, while also taking a possible vehicle effect into account. Hence, they analyzed the 3 treatment groups as independent groups and used multiple t-tests for this analysis (unfortunately without correcting for multiple testing, and in case of seizure latency data testing, only having 3 data points for the Lova-treated group, which is obviously inadequately powered to draw meaningful conclusions). The differences between the 3 treatment groups was subsequently inferred from a comparison of the obtained p-values (significant versus non-significant). Obviously, this method is inadequate (if one p value would be 0.0499 and the other 0.0501, it would have resulted in the erroneous conclusion that the drugs were significantly different), and not in line with the eNeuro spirit to improve rigor and reproducibility, with a strong encouragement to use proper (estimation) statistics.

For our initial submission, we had actually missed that the investigators used different amounts of DMSO when comparing lovastatin with simvastatin. This difference in DMSO amount is indeed a potential problematic issue in the interpretation of the data, since there are reports that DMSO could affect seizure activity. Hence, we agree with the reviewer that our initial analysis was indeed flawed. We have now used a statistical analayis for dichotomous outcomes using a logistic regression model, that also allows us to take the differences in vehicle in account. With this model, we can estimate main effects of a certain predictor to compare the model fit versus a model without that predictor. This analysis showed no hint of a vehicle effect, and further indicated that the lovastatin versus simvastatin treatment are not at all significantly different from each other (Fmr1-lovastatin versus Fmr1-‘high dose’ of simvastatin: p>0.99). Hence, despite the fact that the lovastatin dose was 2-30 fold higher than simvastatin dose, it does not seem to perform significantly better than simvastatin in the seizure assay. We shared the details of this analysis (including the syntax) for verification with Muscas and colleagues.

2. In response to a)the criticism that the drugs were not compared at the same dose---Muscas et al tested a range of simvastatin doses (0.1-5.0 uM) and one effective dose of lovastatin (50uM) in the ERK and protein synthesis assays. The authors found a dose dependent increase in the protein synthesis rates at higher doses of simvastatin (0.5 and 5 uM) which could confound or preclude analysis of very high doses of simvastatin on these Fmr1 KO phenotypes. This should be mentioned by the authors of the commentary.

Response: We agree with the reviewer that the finding that a low dose of simvastatin actually worsened the phenotype, is indeed a very notable finding which was not sufficiently acknowledged by us. We have now emphasized this as follows: ‘The most surprising finding of the study by Muscas and colleagues is the finding that simvastatin treatment at low dose actually worsened the Fmr1 phenotype by further increasing protein synthesis rates. This effect was found to be independent of ERK signaling.’

We were not necessarily implying that the authors should have used a higher dose of simvastatin. Our main point is that the dose of simvastatin and lovastatin should be the same in order to conclude that they have very different effects. In fact, we would really like to know if a lower dose of lovastatin (below the dose needed to inhibit ERK) would have the same stimulating effect on protein synthesis rates. We have now added: ‘It would be of great importance to know if a comparable (low) dose of lovastatin (below the dose needed to inhibit ERK) would have a similar negative effect on this phenotype, especially since the dose that can be safely used in clinical trials is much lower than the in vivo dose used in this study.’

3. The fact that Muscas et al., did show effects of simvastatin on protein synthesis rates, albeit in a distinct direction that lovastatin, indicates the drug was effective. However, because simvastatin did not affect P-ERK at these doses suggests it may be acting through a distinct mechanism from lovastatin, perhaps as a result of its increased efficacy at HMG-CoA reductase or other off target effects. Or lovastatin has off target effects at higher doses on ERK and reduced protein synthesis. This could be considered by the commentary.

Response: We entirely agree with the reviewer that the study may be indicative of the presence of an off-target effect. Importantly, if that is the case, we do not expect the drugs to behave in a linear fashion, which emphasizes the need to perform such experiments at the same dose range for both statins. We have now rewritten this section to make this point clearer, and we explicitly included the possibility of the off-target effect: ‘Muscas and co-workers surprisingly concluded that lovastatin is superior over simvastatin in reducing ERK activation, as well as in its ability to rescue the downstream phenotypes of ERK activation: increased protein synthesis and sensitivity to audiogenic seizures. Given the increased potency of simvastatin to reduce HMG-CoA reductase, it would suggest that the previously demonstrated rescue of RASopathy phenotypes by statins is not mediated by attenuation of RAS farnesylation but rather through an unknown mechanism that is absent or less potent for simvastatin.’

Minor:

1. In doing the estimation of the equivalent doses between human and mice, toward the end of the main text, authors should specify the method they used to perform the dose calculation in both species.

We have added the proper references.

2. It would help to define concepts and gene names such as RAS, PTPN11, HRAS, HMG-CoA, etc.

We have now added the full names.

3. While in another part of the text they mention Neurofibromatosis type I, and they could use NF1 instead.

We have used these terms for mouse models more consistent (i.e. Nf1 mice).
